# TRIM67 Implicates in Regulating the Homeostasis and Synaptic Development of Mitral Cells in the Olfactory Bulb

**DOI:** 10.3390/ijms241713439

**Published:** 2023-08-30

**Authors:** Chunyu Cai, Qihui Luo, Lanlan Jia, Yu Xia, Xinting Lan, Xiaoli Wei, Shuai Shi, Yucong Liu, Yao Wang, Zongliang Xiong, Riyi Shi, Chao Huang, Zhengli Chen

**Affiliations:** 1 Laboratory of Experimental Animal Disease Model, College of Veterinary Medicine, Sichuan Agricultural University, Chengdu 611130, China; callmeccy@163.com (C.C.); lqhbiology@163.com (Q.L.); jialanlan@sicau.edu.cn (L.J.); xiayu113bvs@163.com (Y.X.); lxt9091@163.com (X.L.); wei_xl323@163.com (X.W.); shishuaikeco@163.com (S.S.); liuyuc2021@163.com (Y.L.); aapqwangyao@163.com (Y.W.); loveinchn@outlook.com (Z.X.); 2 Key Laboratory of Animal Disease and Human Health of Sichuan Province, College of Veterinary Medicine, Chengdu 611130, China; 3 Center for Paralysis Research, Department of Basic Medical Sciences, College of Veterinary Medicine, Purdue University, West Lafayette, IN 47907, USA; riyi@purdue.edu

**Keywords:** TRIM67, olfactory bulb, development, mitral cells

## Abstract

In recent years, olfactory dysfunction has attracted increasingly more attention as a hallmark symptom of neurodegenerative diseases (ND). Deeply understanding the molecular basis underlying the development of the olfactory bulb (OB) will provide important insights for ND studies and treatments. Now, with a genetic knockout mouse model, we show that TRIM67, a new member of the tripartite motif (TRIM) protein family, plays an important role in regulating the proliferation and development of mitral cells in the OB. TRIM67 is abundantly expressed in the mitral cell layer of the OB. The genetic deletion of TRIM67 in mice leads to excessive proliferation of mitral cells in the OB and defects in its synaptic development, resulting in reduced olfactory function in mice. Finally, we show that TRIM67 may achieve its effect on mitral cells by regulating the Semaphorin 7A/Plexin C1 (Sema7A/PlxnC1) signaling pathway.

## 1. Introduction

The sense of smell is one of the most basic cognitive functions of living organisms. As an important part of the olfactory system, the olfactory bulb (OB) plays a central role in the processing of olfactory information and is the first relay station between the peripheral and central nervous systems [1]. In mammals, the OB precocious develops in embryo [2]. Two typical neurons, the projection neuron and interneuron, are present in the OB, and projection neurons develop early before interneurons. In mice, projection neurons mainly arise from embryonic day 10.5 (E10.5) to E18, and interneurons’ proliferation mainly begins from E18 and persists to adulthood [3]. Maintaining normal development and biological function of the OB is not only important for the olfactory pathway but also critical for neurological health. Recently, a large number of studies have reported close relationships between olfactory dysfunction and neurological diseases, such as depression, Alzheimer’s disease, and Parkinson’s disease (PD). Patients showing depression and cognitive dysfunction are usually accompanied by symptoms of decreased sense of smell [4,5,6] and decreased neurogenesis in the OB [7], whereas PD leads to a compensatory increase in dopaminergic neurons (a type of interneuron) in the OB [8]. Therefore, understanding the critical molecular mechanisms required for the development and function of the OB will provide a theoretical basis for the diagnosis and treatment of olfactory disorders and related neurological diseases.

Tripartite motif (TRIM) proteins are a large protein family with more than 80 members, which are involved in highly diverse cellular activities and processes like cell proliferation and differentiation, DNA damage response, immune activation, and inflammatory processes [9,10,11]. TRIM67 is a new member of the TRIM protein family, which is much conserved in evolution. The current research on TRIM67 is very limited, most of which focuses on its role in cancer and inflammation. TRIM67 can regulate the progress of nonsmall cell lung cancer and colorectal cancer [12,13], and can inhibit the occurrence of inflammation by inhibiting the activation of NF-kB [14]. In addition, a few studies have reported that TRIM67 is implicated in brain development. Specifically, TRIM67 can interact with TRIM9 to regulate neuronal morphogenesis and function, and loss of TRIM67 leads to impaired spatial memory and cognitive flexibility [15,16,17]. TRIM67 is highly expressed in the OB [18,19], but the role of TRIM67 in the OB is completely unclear. Thus, in this study, with TRIM67 gene knockout mice, we aim to evaluate whether TRIM67 affects the development and function of the OB in mice and to elucidate the underlying mechanism, which may provide new insights into the study of TRIM67 and olfactory disorders.

## 2. Results

### 2.1. TRIM67 Highly Concentrates in the Mitral Cell Layer of the Olfactory Bulb

**Figure 1:**
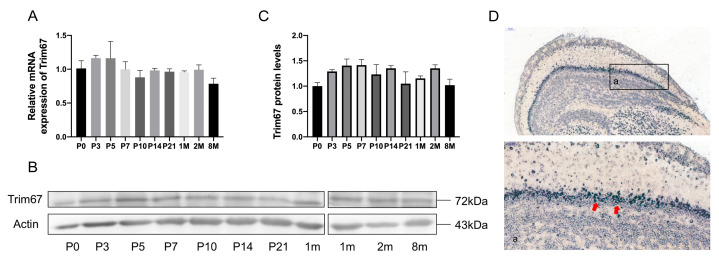
TRIM67 highly concentrates in the mitral cell layer of the olfactory bulb. (**A**–**C**) RT-qPCR and Western blots show the temporal expression pattern of TRIM67 in the postnatal mouse OB (*n* = 3). (**D**) In situ hybridization shows the spatial expression of TRIM67 in the mouse OB. Error bars represent SEM. Red arrows indicate mitral cells.

While TRIM67 is highly expressed in the OB, the function of TRIM67 in the OB is not well defined [18]. To address this issue, we first examined the temporal and spatial expression profiles of *TRIM67* in the mouse olfactory bulb. We observed stable mRNA expression of *TRIM67* in the OB even after birth (Figure 1A), and this was further validated by Western blotting (Figure 1B,C). Furthermore, with in situ hybridization, we found that *TRIM67* mRNA was strongly expressed in the mitral cell layer of the OB (Figure 1D), revealing potential effects of TRIM67 implicated in the function of mitral cells.

### 2.2. TRIM67 KO Affects the Homeostasis of Mitral Cells in the Olfactory Bulb

**Figure 2:**
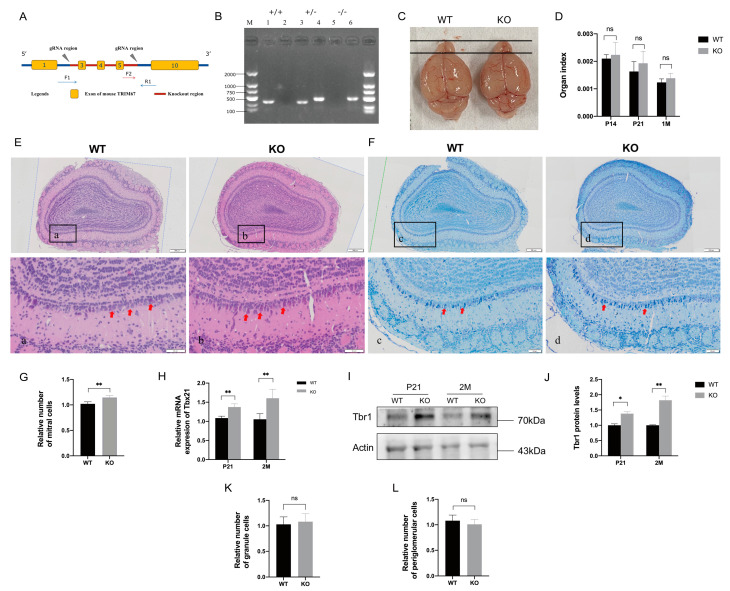
TRIM67 KO affects the homeostasis of mitral cells in the olfactory bulb. (**A**) TRIM67 knockout strategy. (**B**) Genotyping PCR for the validation of TRIM67 knockout mice. (**C**) The picture shows that deletion of the TRIM67 gene does not affect OB size. (**D**) Quantification shows normal organ indices of the OB in TRIM67 KO mice (*n* = 4). (**E**) HE staining show the basic structure in the OB of TRIM67 KO mice. (**F**,**G**) Representative images and quantification of NISSL staining show increased number of mitral cells in the OB of TRIM67 KO mice. (**H**–**J**) RT-qPCR and Western blot show increased expression of mitral cell markers Tbx21 and Tbr1 in the OB of TRIM67 KO mice (*n* = 3). (**K**,**L**) Quantifications reveal that deletion of the TRIM67 gene does not affect the number of granule cells and periglomerular cells in the mouse OB. Error bars represent SEM. * *p*-value < 0.05; ** *p*-value < 0.01. Red arrows indicate mitral cells.

To further investigate the function of TRIM67 in the OB, we constructed a *TRIM67* knockout mouse model (TRIM67 KO) in which exons 3 to 5 of the *TRIM67* gene were deleted (Figure 2A). The genotype of the offspring was assessed by PCR, and KO mice were obtained by mating TRIM67 heterozygous (TRIM67+/−) males with heterozygous females (Figure 2B). We found that the size (Figure 2C) and organ index (Figure 2D) of the OB in TRIM67 KO mice were comparable to those of wildtype (WT) mice. In addition, HE staining (Figure 2E) and NISSL staining (Figure 2F) showed that the structure of OB in TRIM67 KO mice was clear, compact and orderly, the olfactory glomerulus was intact, and the basic structure of the OB was normal. These results suggested that TRIM67 is not required for the basal development of the OB. However, when we focused on mitral cells, where TRIM67 is enriched, we noticed a significant increased number of mitral cells in the OB of TRIM67 KO mice (Figure 2G). Consistently, the mRNA and protein levels of the specific mitral cell markers, Tbx21 and Tbr1, were also increased (Figure 2H–J). In contrast, no changes in the number of granule cells and periglomerular cells were observed (Figure 2K,L). Collectively, these results suggested that TRIM67 was required for the homeostasis of mitral cells in the OB.

### 2.3. TRIM67 Affects the Proliferation of Mitral Cells in Embryonic Mouse

**Figure 3:**
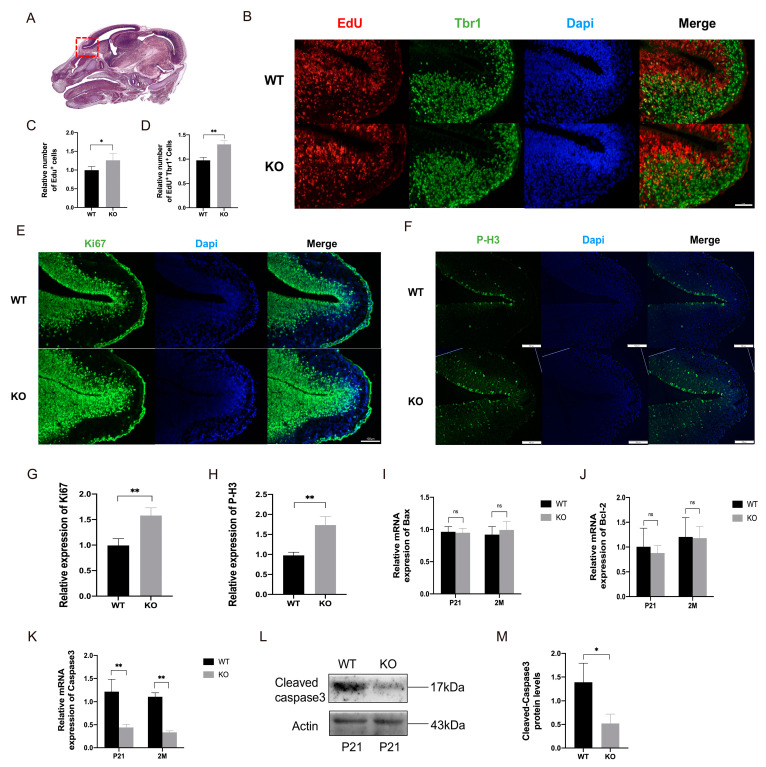
TRIM67 affects the proliferation of mitral cells in an embryonic mouse. (**A**) Schematic diagram of the OB in E14 mice [23]. The red dotted area represents the OB. (**B**–**D**) Immunostaining and quantifications of Edu and Tbr1 reveal an increased number of newly proliferating mitral cells in the OB of E14 TRIM67 KO mice (*n* = 3). (**E**–**H**) Immunostaining and quantifications of Ki67 and P-H3 reveal that the proliferation in OB cells of E14 TRIM67 KO mice was enhanced (*n* = 3). (**I**–**M**) RT-qPCR and Western blot show reduced apoptosis in the OB of TRIM67 KO mice (*n* = 4). Error bars represent SEM. * *p*-value < 0.05; ** *p*-value < 0.01.

While multiple factors affect the number of matured neurons, the homeostasis of cellular proliferation and death plays important roles [20]. The OB is located on the rostral side of the forebrain, as shown in Figure 3A, and develops from the embryonic stage. Mitral cells are the first type of neurons to appear during the development of the OB, and mainly proliferate during E10.5 to E15. To understand how TRIM67 affects mitral cells, Edu/Tbr1 double-labeling was first performed to quantify the generation of mitral cells in the OB during E12 to E14 (the peak period of mitral cell proliferation) (Figure 3B). We found that both the number of the Edu positive cells and Edu/Tbr1 positive cells were increased in TRIM67-deficient OBs, indicating enhanced proliferation rate of OB cells and especially mitral cells (Figure 3C,D). Consistently, immunofluorescence labeling of KI67 and PH-3, two common markers for cell proliferation [21], also displayed increased cellular proliferation in the OB of TRIM67 KO mice (Figure 3E,H). Previous reports have shown that TRIM67 is involved in the regulation of apoptosis [13,18,22], which may also affect tissue homeostasis. Interestingly, with RT-qPCR and Western blot, we found no changes in the expressions of apoptotic factors Bax and Bcl-2, while the expressions of major apoptotic executor caspase-3/cleaved-caspase-3 were significantly suppressed in the OB of TRIM67 KO mice. Therefore, these results revealed that TRIM67 deletion increased the number of mitral cells in the OB by promoting cell proliferation.

### 2.4. Loss of TRIM67 Causes Olfactory Dysfunction in Mice

**Figure 4:**
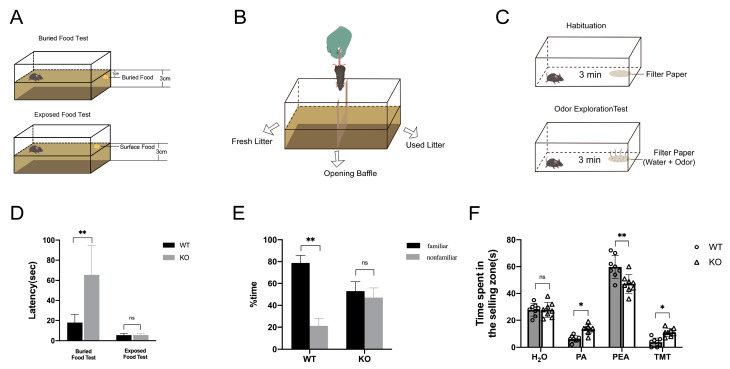
Loss of TRIM67 causes olfactory dysfunction in mice. (**A**–**C**) Schematic diagrams showing the food burial, olfactory discrimination, and odor exploration experiments. (**D**) Statistical analysis of the time for WT and KO mice to find hidden or exposed food particles in the food burial and the food exposure tests (*n* = 8). (**E**) Statistical analysis of time spent in familiar and unfamiliar odor environments by WT and KO mice (*n* = 8). (**F**) Statistical analysis of odor exploration time for H_2_O, PA, PEA and TMT in WT and KO mice (*n* = 8). Error bars represent SEM. * *p*-value < 0.05; ** *p*-value < 0.01.

Affected homeostasis of mitral cells in the OB always results in olfactory dysfunction. To evaluate whether increased mitral cells after TRIM67 deletion affect the sense of smell in mice, we conducted three different behavioral tests: food burial, olfactory discrimination, and odor exploration. In the food burial experiment, TRIM67 KO mice took significantly longer time to find food than WT mice, whereas in the food exposure experiment, which served as a control experiment, TRIM67 KO mice took a comparable amount of time to find food as WT mice, indicating that the perception and exploration ability of TRIM67 KO mice was weakened (Figure 4D). In the olfactory discrimination test, WT mice could discriminate between familiar and unfamiliar odors and stayed longer in familiar spaces, while TRIM67 KO mice could not discriminate and spent the same amount of time in familiar and unfamiliar spaces, showing defects in the discrimination ability of TRIM67 KO mice (Figure 4E). In the odor exploration experiment, WT mice and TRIM67 KO mice spent comparable time exploring odorless double distilled water, but TRIM67 KO mice took longer time than WT mice to explore aversive odors (Propanoic acid, PA; Trimethylthiazoline, TMT), and spent less time exploring attractive odors (Phenethylamine, PEA), showing reduced olfactory sensitivity in TRIM67 KO mice (Figure 4F). These results collectively revealed that loss of TRIM67 caused olfactory dysfunction in mice.

### 2.5. Impaired Synapse Formation in Mitral Cells of TRIM67 KO Mice

**Figure 5:**
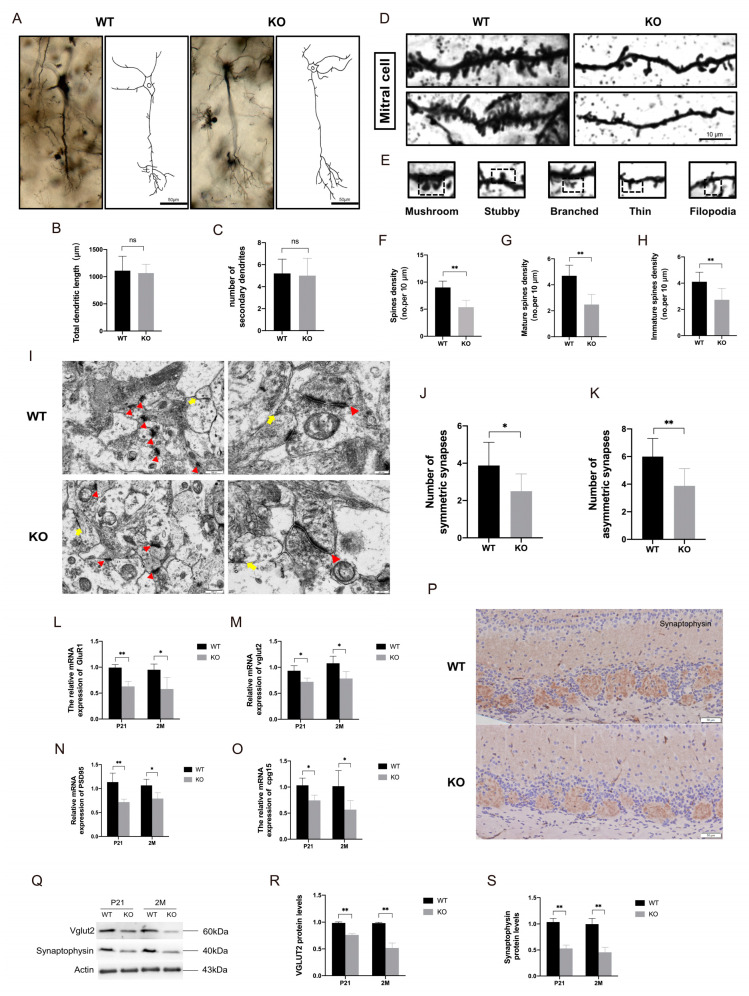
Impaired synapse formation in mitral cells of TRIM67 KO mice. (**A**–**C**) Representative images of Golgi-stained mitral cells and statistical analysis of dendrites show normal development of mitral cell dendrites in the OB of TRIM67 KO mice (*n* = 3). (**D**,**E**) Representative images of Golgi-stained dendritic spines of mitral cells and schematic diagrams of various types of dendritic spines. (**F**–**H**) Statistical analysis of mature, immature, and total dendritic spine numbers show decreased dendritic spines in mitral cells in TRIM67 KO mice (*n* = 3). (**I**–**K**) Electron microscopy and quantification of representative images of synapses in the OB show a decrease in the number of synapses in the OB of TRIM67 KO mice (*n* = 3). Red arrows indicate asymmetric synapses, yellow arrows indicate symmetric synapses. (**L**,**M**) RT-qPCR reveal decreased expression of synapse-associated markers in the olfactory bulb of KO mice compared with WT mice (*n* = 4). (**N**,**O**) RT-qPCR reveal that the expressions of factors that promote synapse development were decreased in the olfactory bulb of KO mice compared with WT mice (*n* = 4). (**P**–**S**) Immunostaining, Western blot, and quantification show decreased protein expression of VGLUT2 and Synaptophysin in the olfactory bulb of KO mice compared with WT mice (*n* = 4). Error bars represent SEM. * *p*-value < 0.05; ** *p*-value < 0.01.

These findings are interesting in that increased mitral cells are accompanied by impaired olfactory function. Therefore, to evaluate the basis underlying this phenotype, we first analyzed the morphological structure of mitral cells through Golgi staining, and found that deletion of TRIM67 had no effect on the overall morphology of mitral cells (Figure 5A). Quantifications also showed that the total length of dendrites and the number of secondary dendrites of mitral cells in TRIM67 KO mice were comparable to those of WT mice (Figure 5B,C). Dendritic spines are tiny protrusions located on the dendrites of neurons, which play a huge role in the transmission and communication of information [24]. We further quantified the dendritic spines of mitral cells and surprisingly found that the density of dendritic spines was significantly reduced in TRIM67 KO mice (Figure 5D). Dendritic spines can be divided into mature dendritic spines (mushroom-shaped) and immature dendritic spines (stubby, branched, thin and filopodia-shaped) according to morphological differences [25] (Figure 5E). Quantifications showed that dendritic spine density of mitral cells in TRIM67 KO mice decreased, including mature dendritic spine density and immature dendritic spine density (Figure 5F–H). Dendritic spines are the transmission sites of excitatory synapses, capable of receiving information and forming synaptic connections, and are considered to be the basis for synaptic plasticity [26]. Abnormal dendritic spine formation and pruning may lead to alterations in synaptic structure and function [27,28]. Therefore, we further used electron microscopy to analyze the synaptic morphology and structure in the OB. As shown in Figure 5I–K, we observed a reduced number of asymmetric synapses (excitatory synapses) and symmetric synapses (inhibitory synapses) in the OB of TRIM67 KO mice. Moreover, this synapse defect was further validated by RT-qPCR, immunostaining, and immunoblotting of common synaptic markers (vesicular glutamate transporter type 2, vGLUT2; glutamate receptor 1, GluR1; Synaptophysin) and synaptic maturation factors (candidate plasticity-related gene 15, *cpg15*; Postsynaptic density protein 95, PSD95) (Figure 5L–S). All these data suggested that deletion of TRIM67 led to impaired synapse formation in mitral cells, which should be responsible for olfactory dysfunction.

### 2.6. Sema7A/PlxnC1 Signaling Implicates in TRIM67-Regulated Synapse Development of Mitral Cells

**Figure 6:**
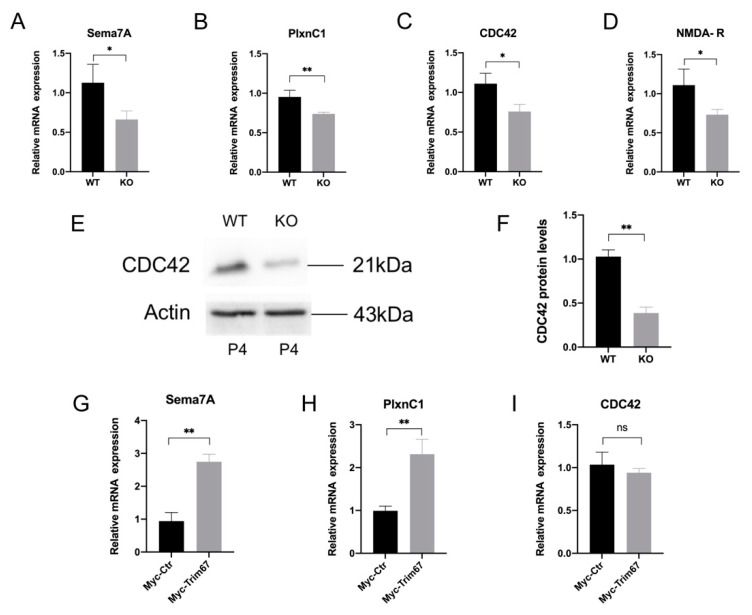
Sema7A/PlxnC1 signaling implicates in TRIM67-regulated synapse development of mitral cells. (**A**) RT-qPCR reveals that the expression level of Sema7A mRNA was decreased in the OB of KO mice compared with WT mice (*n* = 4). (**B**) RT-qPCR reveals that the expression level of PlxnC1 mRNA was decreased in the OB of KO mice compared with WT mice (*n* = 4). (**C**) RT-qPCR reveals that the expression level of CDC42 mRNA was reduced in the OB of KO mice compared with WT mice (*n* = 4). (**D**) RT-qPCR reveals that the expression level of NMDA-R mRNA in the OB of KO mice was reduced compared with that of WT mice (*n* = 4). (**E**,**F**) Western blot and quantification show reduced protein expression of CDC42 in the OB of TRIM67 KO mice (*n* = 3). (**G**–**I**) RT-qPCR quantifications show increased expression of genes related with the Sema7A/PlxnC1 signaling pathway in TRIM67 overexpressed 293T cells (*n* = 4). Error bars represent SEM. * *p*-value < 0.05; ** *p*-value < 0.01.

The signaling protein Sema7A is a key regulator of axon guidance that assists growing axons in finding appropriate targets and forming synapses [29]. A previous study reported that the interaction between Sema7A and PlxnC1 can regulate synapse formation in mitral cells [30]. Therefore, we further investigated a possible connection between the Sema7A/PlxnC1 pathway and the synapse defects in mitral cells in Trim67 KO mice. Since the Sema7A/PlxnC1 pathway mainly plays a role at postnatal day 3–5 (P3–P5), we analyzed these factors in the OB of P4 mice. We found that compared with the control mice, the mRNA levels of Sema7A, PlxnC1, cell division cycle 42 (CDC42) and N-methyl-D-aspartate receptor (NMDA-R), which are key factors regulating mitral cell synapse development in the Sema7A/PlxnC1 pathway, were decreased in the OB of TRIM67 KO mice (Figure 6A–D). Western blotting also showed that CDC42 protein expression was suppressed in TRIM67 KO mice (Figure 6E,F). In contrast, we found that TRIM67 overexpression promoted the expressions of Sema7a and PlxnC1 in cultured cells, with no changes in CDC42 expression (Figure 6G–I). Therefore, the correlated expression pattern of TRIM67 with Sema7A, PlxnC1 suggested that loss of TRIM67 may inhibit synaptic development in mitral cells by inhibiting the Sema7A/PlxnC1 signaling pathway.

## 3. Discussion

The OB is the key center for smell perception, while mitral cells, as projection neurons in the OB, play a vital role in the process of olfactory information transmission. Although the developmental process of mitral cells has been well described, the complex molecular mechanisms behind it have yet to be elucidated. In this study, we report that TRIM67 is a key protein that regulates the proliferation and development of OB mitral cells. TRIM67 is highly expressed in the OB, mainly concentrated in mouse mitral cells, and participates in the regulation of mitral cell proliferation and synapse development. Loss of TRIM67 in mice leads to abnormal proliferation of mitral cells in the OB and impairs synapse development, resulting in olfactory dysfunction.

TRIM32 is homologous to TRIM67. Previous research by Hillje et al. clarified that the absence of TRIM32 leads to the overproliferation of adult-generated OB neurons, thereby causing diminished olfactory function [31,32]. In the current study, we found that TRIM67 has a similar role in the OB. The difference is that their study focused on the proliferation of adult-generated OB interneurons, while we focused on mitral cells. This is due to the differential expression patterns of TRIM32 and TRIM67 in the OB, in which TRIM32 is upregulated during the differentiation of adult neural stem cells into OB neurons, whereas TRIM67 is stably expressed in mitral cells. In addition, although both studies showed that increased OB neurons lead to decreased olfactory function, they did not delve into the reason for the dysfunction of TRIM32-deficient OB neurons, but rather speculated on the reasons for this phenotype from the perspective of imbalance in metabolite concentrations in brain tissue. We, in contrast, have investigated in detail the morphology and function of mitral cells and found that diminished olfactory function is caused by defective synaptic development in mitral cells.

Our study shows that TRIM67 has certain potential in neuronal proliferation, which has been supported in a previous study [17]. Interestingly, TRIM67 is also involved in regulating the proliferation of cancer cells, and plays various roles in different cancers. In colorectal cancer, TRIM67 inhibits cancer cell proliferation [13,33], whereas in nonsmall cell lung cancer it promotes cancer cell proliferation [12]. The above results show that the trend of TRIM67’s role in cell proliferation is not completely consistent, and it may have completely opposite effects in different tissue or cellular conditions, and the specific mechanism behind it remains to be explored. Similarly, the role of TRIM67 in apoptosis is also inconclusive. Our study and some previous studies show that TRIM67 can promote apoptosis [13,22], but other researchers found that TRIM67 can also inhibit apoptosis [18,34]. We still do not understand the reason for this phenomenon, but the mechanism behind it is well worth exploring.

Another interesting finding reported by our study is that declined olfactory function correlated with increased mitral cells. It is known that dendritic spines are one of the basic elements of synapses, and form complete synaptic connections with projected axons. The morphology and structure of dendritic spines are characterized by significant plasticity, and changes in dendritic spine morphology and structure often lead to alterations in synaptic function [35,36]. We found that the number of dendritic spines and synapses was reduced in the OB mitral cells of TRIM67 KO mice, which explained well the phenotype of diminished olfactory function in TRIM67-deficient mice. Regarding the role of TRIM67 in the nervous system, previous studies have shown that TRIM67 is involved in promoting neuronal morphogenesis, pruning neural connections, and regulating axon guidance [16,37,38,39,40]. In this study, although the specific effect of TRIM67 on the neurites of mitral cells is not exactly the same as reported, there is a certain correlation. It is clear that TRIM67 is essential for the development and maturation of neuronal neurites. In addition, inflammatory responses in the central nervous system can also lead to synapse loss [41], and TRIM67 has been shown to be associated with inflammatory responses. To address whether the synaptic reduction caused by TRIM67 deletion was correlated with inflammation, we examined the relevant inflammatory factors in the olfactory bulb (Figure S1) and found that TRIM67 knockdown did not produce an inflammatory response in the olfactory bulb, which further clarified that synaptic damage in TRIM67 KO mice was caused by incomplete development of the mitral cells.

Our work also provides further information for the underlying mechanism by which TRIM67 regulates synapse development in mitral cells. Sema7A/PlxnCl signaling has been reported to play an important role in early synapse development in mitral cells [30]. In this study, we found that TRIM67 deletion suppressed PlxnCl expression and simultaneously altered the expression trend in related molecules upstream and downstream of PlxnCl, which suggested that TRIM67 may regulate synapse development of mitral cells through this pathway. Moreover, the formation of dendritic spines in mitral cells is also closely related to this pathway. The stabilization of actin filaments in dendritic spines is regulated by CDC42 molecules in this signaling pathway [42], and the overexpression of CDC42 in neurons rescued the reduction in dendritic spines caused by Axin deficiency [43]. Therefore, we hypothesized that TRIM67 simultaneously regulates the development of dendritic spines and synapses in mitral cells through this pathway. More interestingly, overexpression of TRIM67 promoted the expression of Sema7A and PlxnC1 in vitro, but had little effect on CDC42. Therefore, we further speculated that TRIM67 may mainly act on the upstream of this signaling pathway. Specifically, as proteins coexpressed in mitral cells, TRIM67 and PlxnC1 may interact to regulate this signaling pathway. However, there is still a lack of strong evidence to prove this conjecture. Although we found that TRIM67 is associated with the Sema7A/PlxnC1 signaling pathway, the specific mechanism of its regulation in the signaling pathway is still unclear, which is also the focus of our next research.

## 4. Materials and Methods

### 4.1. Animals

All the mice were treated according to Sichuan Agricultural University’s Animal Care and Use Committee guidelines. The mouse strain used in this study is C57BL/6N. Conventional TRIM67 knockout mice (TRIM67 −/−, KO) were produced by Cyagen Biosciences (Suzhou, China) using the CRISPR-Cas9 system targeting exons 3 to 5 of the TRIM67 gene (Figure 2A). The target sequence of the gRNA are as follows: gRNA1 (corresponding forward strand of the gene): TCT GGG TAG GTA ACG GCT TCT GG; gRNA2 (corresponding reverse strand): CAG GCT CAA GGG GGT CTA GAC GG. PCR (WT Forward: 5′-GAT GAT AGC CAT GTA ATG CCC ACC-3′, Reverse: 5′-CCG TGA TAT GCT TGC CAC AGG TTC-3′, Reverse: 5′-TTG ATG GTT GG AGC CCT GC-3’) were used to identify founder mice and their progeny. The PCR assay was performed on the genomic DNA isolated from tail cuttings. Under SPF conditions, all mice were kept in ventilated cages at 20–22 °C, 12 h light/12 h dark cycle, 50–70% humidity, and access to standard food and water.

### 4.2. RT-qPCR

Total RNA was extracted from tissues with the Animal Total RNA Isolation Kit (RE-03014, Foregene, Chengdu, China). Then, RT EasyTM II (with gDNase) (RT-01032, Foregene, Chengdu, China) was used to reverse transcription using ~1 µg RNA. The Bio-Rad CFX96 system (Bio-Rad, Hercules, CA, USA) was used for RT-qPCR, and the related gene expression was standardized to β-Actin. The primers were used in this study are listed in Table 1.

### 4.3. Western Blots

The proteins from olfactory bulb tissues were extracted by the RIPA lysis buffer system, and SDS-PAGE was performed to isolate the whole proteins in all groups. After transferring the proteins onto the PVDF film (Millipore, Darmstadt, Germany), a 1 h block in TBST (containing 5% skimmed milk) was taken. The film was then incubated at 4 °C overnight with a primary antibody (Table 2). After washing with TBST, the film was incubated for 1 h with a second antibody (1:10,000; Absin). Lastly, the Western Blot Imaging System (ChemiDoc MP, Bio-rad, Hercules, CA, USA) was used to detect and scan the protein signals. The TRIM67 antibody was generated by immunizing rabbits.

### 4.4. Histological Staining

The mouse olfactory bulbs were fixed in 4% PFA solution, embedded in paraffin, and then cut into 5 μm slices. The slices were stained with HE and NISSL according to the manual (G1120 for HE and G1432 for NISSL; Solarbio, Beijing, China).

### 4.5. Immunohistochemical Staining

The specific experimental steps were carried out according to the SADB-POD kit manual (Boster, SA2002, Wuhan, China). The paraffin sections were dewaxed first, and then blocked with 3% hydrogen peroxide for 20 min. After washing with PBS, antigen retrieval was performed with sodium citrate buffer. Next, the sections were blocked (blocking buffer), incubated with primary antibody (4 °C, overnight), and incubated with secondary antibody (1 h), in sequence. After washing with PBS, the slides were incubated with SABC (30 min). Finally, color development was carried out with DAB and the slides were stained with hematoxylin.

### 4.6. Immunofluorescence Staining

Immunofluorescence paraffin sections were first deparaffinized and rehydrated, followed by high-pressure repair with sodium citrate buffer. Then, the sections were blocked with blocking buffer for 1 h (10% donkey serum in PBS+ 0.1% Triton X-100), incubated with primary antibody (4 °C, overnight), and incubated with secondary antibody (1 h), in sequence. After washing with PBS, the slides were mounted with the DAPI-containing mounting medium (Thermo, P36962, Waltham, MA, USA).

### 4.7. EDU Labeling Experiment

The specific experimental steps were carried out according to the EDU labeling kit manual (Beyotime, C0078S, Shanghai, China). Briefly, the female mice that detected the vaginal plugs were recorded as E0.5. At E12, the pregnant mice were intraperitoneally injected with EdU working solution (50 mg/kg), for two consecutive days for a total of 4 times, and the brains of embryonic mice were taken at E14 and fixed in paraformaldehyde. Paraffin sections were made according to routine procedures and immunofluorescence staining was performed, and then the prepared Click reaction solution was added dropwise to the tissue and incubated for 30 min. After washing, the slices were mounted with DAPI-containing mounting medium (Thermo, P36962, Waltham, MA, USA).

### 4.8. Golgi Staining

The specific experimental steps were carried out according to the Golgi staining kit manual (Servicebio, Wuhan, China). Briefly, the olfactory bulbs were taken and immediately rinsed gently with saline, then placed in EP tubes, submerged in Golgi staining solution, and left in a cool, ventilated, and dark place for 14 days (the staining solution was changed every three days). After 14 days, the distilled water-rinsed tissues were first placed in 80% glacial acetic acid until the tissues became soft, and then in 30% sucrose solution to dehydrate. Next, the olfactory bulbs were cut into 100 μm, and the sections were left to dry overnight in the dark. Finally, the sections were subjected to 15 min treatment with concentrated ammonia and 1 min wash with distilled water. Afterward, the sections were treated with acidic firm film fixative for 15 min, washed with distilled water for 3 min, dried, and sealed with glycerol gelatin.

### 4.9. Electron Microscope Experiment

Fresh tissue samples were put into electron microscope fixative (Servicebio, G1102, Wuhan, China). The tissues were rinsed with 0.1 M phosphate buffer (pH 7.4) for 3 times (15 min for each), and then fixed with 1% osmic acid-0.1 M phosphate buffer for 2 h. The fixed tissues were rinsed again with 0.1 M phosphate buffer and then dehydrated in gradient alcohol and acetone in turn. The tissues were then embedded by infiltration with acetone and 812 embedding agent (acetone: 812 embedding agent = 2:1 infiltration overnight, pure 812 embedding agent for 5–8 h). The pure 812 embedding agent was poured into the embedding plates, and then the samples were inserted into the embedding plates and placed in the oven at 37 °C overnight. The embedding plates were placed in an oven at 60 °C for 48 h of polymerization and the resin blocks were taken out for later use. Ultrathin sections of 60–80 nm were taken from the tissue with an ultramicrotome, and then the slices were picked up with a 150-mesh square film copper net. The copper nets were stained in 2% uranyl acetate saturated alcohol solution in the dark for 8 min, washed 3 times with 70% alcohol, washed 3 times with ultrapure water, and stained with 2.6% lead citrate solution for 8 min away from carbon dioxide. The copper mesh slices were placed in a copper mesh box to dry overnight at room temperature. The tissues were observed under a transmission electron microscope and images collected for analysis.

### 4.10. Food Burial Experiment

Specific steps referred to previous work [44]. Before the experiment, the body weight of the mice was measured and recorded, food intake was restricted (0.2 g per day for 3 consecutive days), and water was allowed freely for drinking. The mice were acclimatized to the test room for 1 h before the experiment on day 4. Mice were placed in an opaque test cage, and food pellets (about 2 g in weight) were buried at a depth of 3 cm below the litter. The mice were placed in the corner farthest from the burial point of the food pellets, and then the timing was started from the time the mice were placed in the cage and stopped when they grasped the food pellet with their front paws or teeth; if the mice did not find the food within 5 min, the recording time was 300 s. As a control, a visible food pellet test was finally carried out. The food pellets were placed in the middle of the bedding surface of the cage, and the time from when the mice were placed in the cage to when they found the food was recorded.

### 4.11. Odor Probing Test

Specific steps referred to previous work [45]. Mice were reared in single cages and deprived of water for 48 h, and then placed in clean and disinfected cages. Before the experiment, a clean filter paper was put in the corner of the mouse cage and the mice were allowed to adapt for 3 min; 10 μL of different liquids was then added to the center of the filter paper in sequence: dipure water, 10% propionic acid solution (PA), 2% trimethylthiazoline solution (TMT), 10% phenethylamine solution (PEA). The mice were then placed in the cage to explore freely for 3 min, and a camera system was used to record and track the movement trajectory and exploration time of the mice. When a mouse’s head first entered the filter paper at 1 cm, the time recording was started and the total time of the mouse’s head exploration in the filter paper was recorded.

### 4.12. Odor Discrimination Test

Specific steps referred to previous work [46]. Each mouse was placed separately in a cage for 5 min, and the cage was divided into two identical compartments by an open door. One compartment was filled with fresh bedding (unfamiliar), while the other compartment was filled with old bedding (familiar). The time (seconds) that the mice spent in each of the two compartments (familiar and unfamiliar) were recorded.

### 4.13. Cell Culture

Specific 293T cells were obtained from the National Cell Line Resource Infrastructure (Beijing, China). Cells were cultured in DMEM medium containing 10% FBS (P30-3302, PAN, Adenbach, Germany) and 1% penicillin–streptomycin solution (P1400, Solarbio, Beijing, China). Empty plasmid and PRK5-MYC-TRIM67 plasmid were transfected into cells using transfection reagents (LipoMax TM (32012, SUDGEN, Nanjing, China) and opti-MEM (31985070, Gibco), respectively). Twenty-four hours after transfection, cells were harvested for RT-qPCR assays.

### 4.14. Statistical Analysis

All data were presented as mean ± standard error of the mean (SEM). Two-tailed Student’s *t* test or one-way ANOVA was performed for the statistical significance analysis using GraphPad Prism software (Version 6.0, San Diego, CA, USA); * *p* < 0.05, ** *p* < 0.01.

## 5. Conclusions

In summary, this study reveals that TRIM67 is involved in the regulation of mitral cell development in the mouse olfactory bulb. Loss of TRIM67 in the olfactory bulb leads to hyperproliferation of mitral cells and defects in synapse development in mice. Additionally, we show that TRIM67 regulates the development of mitral cells through the Sema7A/PlxnC1 signaling pathway.

## Figures and Tables

**Table 1:** The primers used for the RT-qPCR analysis.

Gene	Sequence (5′-3′)
*β-actin*	*F*: AGAGGGAAATCGTGCGTGAC
	*R*: CAATAGTGATGACCTGGCCGT
*TRIM67*	*F*: GGCGAAGGAGTTTCTGGTTC
	*R*: TAGCTTCAGGGTGCAGTGATT
*TNF-α*	*F*: ACGGCATGGATCTCAAAGAC
	*R*: GTGGGTGAGGAGCACGTAG
*IL-21*	*F*: TCAAGCCATCAAACCCTGGA
	*R*: CATACGAATCACAGGAAGGGC
*Tbx21*	*F*: TGTTCCCAGCCGTTTCTACC
	*R*: GCTCATCTTGGGCGGGTATT
*Bax*	*F*: TGGAGATGAACTGGACAGCA
	*R*: TGAAGTTGCCATCAGCAAAC
*Bcl-2*	*F*: AGGATTGTGGCCTTCTTTGA
	*R*: CAGATGCCGGTTCAGGTACT
*Caspase3*	*F*: TGGAGAAATTCAAAGGACGGG
	*R*: AGCATGGACACAATACACGGG
*PSD95*	*F*: CAATGGTGTTGACCTCCGCA
	*R*: GGGGTTGCTTCGCAGAGAT
*VGLUT2*	*F*: GGATCGTTCTTCTGGGGCTAT
	*R*: GTAGAGGTGAGCAGTATCGCA
*cpg15*	*F*: GGGCAAGTGTGATGCAGGTCT
	*R*: TCCTGGCAATCCGTAAGAGC
*GluR1*	*F*: CCCGTTGACACATCCAATCAG
	*R*: GGTATCCTTCCTCCGTGGTT
*Sema7A*	*F*: TGCGGGTAGCGAAGGTTTTC
	*R*: CATGGTCCTGCCCTTTCCAG
*PlxnC1*	*F*: CAAGGGGATTGCACACATGCT
	*R*: GGTCCAGTTCAGTTGGCTTC
*Cdc42*	*F*: GGAGAGCCATACACTCTTGGAC
	*R*: ATGAGGATGGAGAGACCACTGA
*NMDA-R*	*F*: AACGACCACTTCACTCCCAC
	*R*: CGCGCATCATCTCAAACCAG
*Iba1*	*F*: CTTTTGGACTGCTGAAGGC
	*R*: GTTTCTCCAGCATTCGCTTC

**Table 2:** Antibodies used in this study.

Antibodies	Source	Catalog No./Dilution
Tbr1 Rabbit pAb	Proteintech	20932-1-AP/WB 1:1000
Iba1 Rabbit mAb	Abcam	ab178846/WB 1:1000
Cleaved-caspase3 Rabbit pAb	CST	#9664/WB 1:1000
VGLUT2 Rabbit mAb	Sigma	ZRB1170/WB 1:1000
Synaptophysin Rabbit mAb	CST	#36406/WB 1:1000
CDC42 Rabbit pAb	Proteintech	10155-1-AP/WB 1:1000

## Data Availability

Source data are provided in this paper and are available from the corresponding author upon reasonable request.

## Supplementary Materials

The following supporting information can be downloaded at: https://www.mdpi.com/article/10.3390/ijms241713439/s1.
