# Supplementary material for: TRIM67 Implicates in Regulating the Homeostasis and Synaptic Development of Mitral Cells in the Olfactory Bulb
Source: Int J Mol Sci. 2023 Aug 30;24(17):13439. doi: 10.3390/ijms241713439 (PMC10487898; doi:10.3390/ijms241713439)
Supplement: Supplementary file 1 [file ijms-24-13439-s001.zip › ijms-2572091-supplementary.pdf]

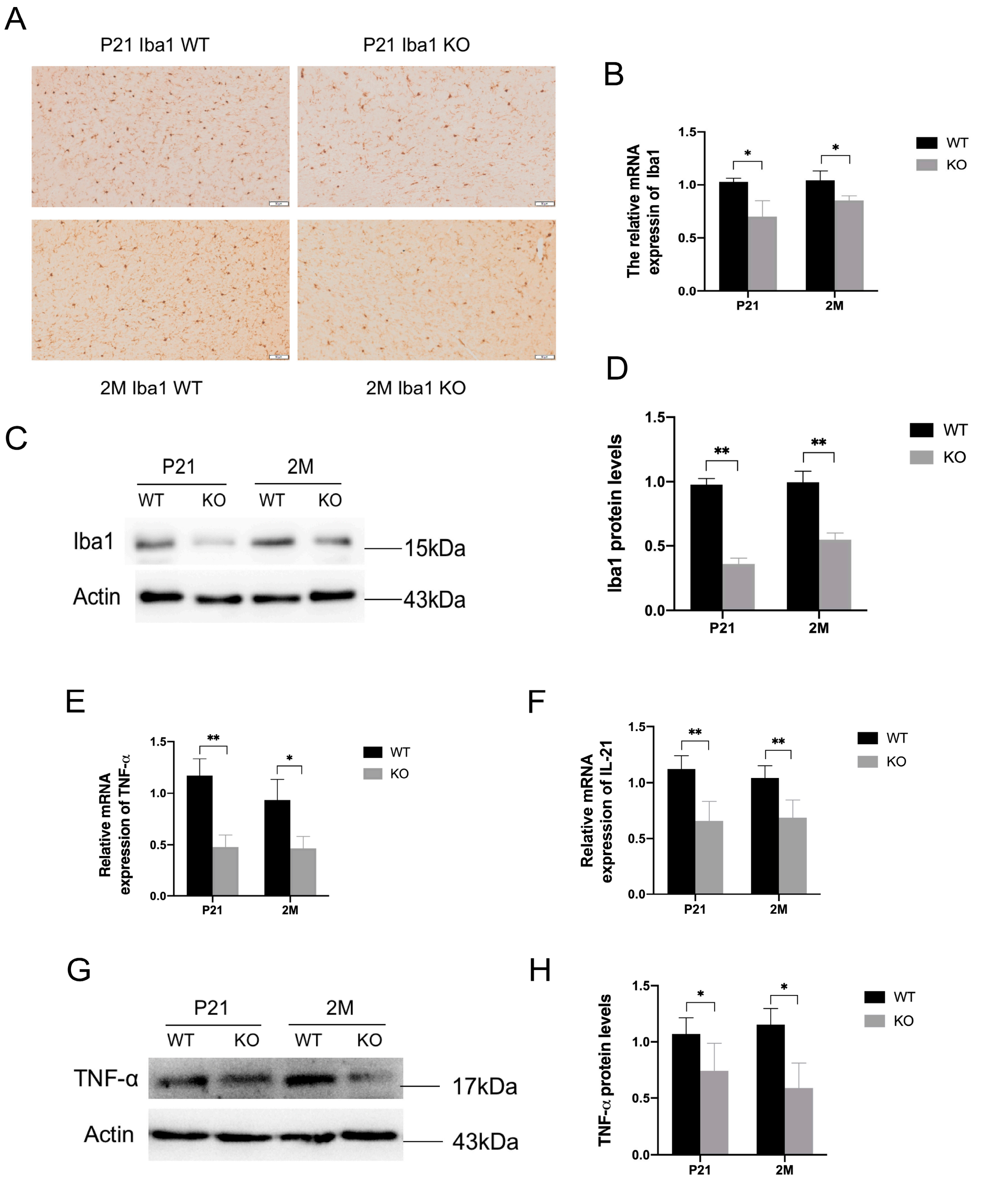

Figure S1: TRIM67 deficiency exerts a protective effect against inflammation in the mouse olfactory bulb. (A–D) Immunostaining, RT-qPCR, Western blots, and quantification of Iba1 reveal reduced microglial activation in the OB of TRIM67 KO mice (n = 3). (E–H) RT-qPCR, Western blots, and quantification of TNF-α and IL-21 show reduced inflammatory response in TRIM67 KO mice (n = 3). Error bars represent SEM. * p-value < 0.05; ** p-value < 0.01.
